# Contrasting stability of fungal and bacterial communities during long-term decomposition of fungal necromass in Arctic tundra

**DOI:** 10.1186/s40793-025-00730-5

**Published:** 2025-06-20

**Authors:** Andrea Moravcová, Florian Barbi, Camelia Algora, Gabriele Tosadori, Petr Macek, Jana Albrechtová, Petr Baldrian, Petr Kohout

**Affiliations:** 1 Laboratory of Environmental Microbiology, Institute of Microbiology of the Czech Academy of Sciences, Prague 4, Czech Republic; 2 Department of Experimental Plant Biology, Faculty of Science, Charles University, Viničná 5, Prague, 128 404 Czech Republic; 3 Laboratorio de Biodiversidad y Funcionamiento Ecosistémico, Instituto de Recursos Naturales y Agrobiología de Sevilla (IRNAS), CSIC, 41013 Seville, Spain; 4 Biology Centre of the Czech Academy of Sciences, Institute of Hydrobiology, Na Sadkach 7, Ceske Budejovice, 370 05 Czech Republic; 5 Department of Biodiversity and Nature Tourism, Institute of Agricultural and Environmental Sciences, Estonian University of Life Sciences, Kreutzwaldi 5a, 51006 Tartu, Estonia

**Keywords:** Fungal necromass, Decomposition, Arctic tundra, Fungal communities, Bacterial communities

## Abstract

Decomposition is a crucial process in terrestrial ecosystems, driving nutrient cycling and carbon storage dynamics. Considering the amount of fungal necromass produced in soils annually, its decomposition represents an important nutrient recycling process. Understanding the decomposition dynamics and associated microbial communities of fungal necromass is essential for elucidating ecosystem functioning, especially in environmentally sensitive regions such as the Arctic tundra, which remain under-explored. In a three-year field experiment conducted in the Svalbard archipelago, we investigated the decomposition of two types of fungal necromass with differing biochemical properties. We studied the decomposition rate, changes in chemical composition, and the succession of fungal and bacterial communities associated with the decaying fungal necromass. We discovered that up to 20% of fungal necromass remained even after three years of decomposition, indicating that the decomposition process was incomplete. Our results indicate the crucial role of *Pseudogymnoascus* in decomposing low-quality, highly melanized necromass with a high C:N ratio in Arctic soils, underscoring its importance in carbon cycling in the Arctic tundra. Notably, we observed dynamic changes in bacterial communities, with increasing richness over time and a shift from copiotrophic to oligotrophic species specializing in decomposing recalcitrant material. Our study indicates the strong potential that fungal necromass can play in carbon sequestration of arctic soils and reveals the distinct dynamics between rather stable fungal and rapidly changing bacterial communities associated with the decomposing fungal necromass in the Arctic tundra. These findings enhance our understanding of microbial succession during decomposition in extreme environments and highlight the potentially differing roles of fungi and bacteria in these processes.

**Supplementary Information:**

The online version contains supplementary material available at 10.1186/s40793-025-00730-5.

## Introduction

Soils are the largest carbon reservoir on Earth’s land, storing more carbon than the vegetation and the atmosphere combined. This terrestrial soil carbon stock peaks at high latitudes, in boreal and Arctic regions where decomposition is slower compared to warmer regions [16, 56]. The importance of plant and microbial necromass in the formation of soil organic matter remains unclear and probably differs among various ecosystems. Accumulation of soil organic matter in high latitudes is primarily driven by plant root activity and their associated microorganisms, particularly fungi [14, 36], whose necromass can represent a substantial proportion of soil carbon [26, 24]. High-latitude ecosystems and the carbon sequestered in their soils are particularly vulnerable to the impacts of ongoing climate change, especially as they are warming much faster than the Earth on average [59]. Understanding the decomposition process of microbial necromass and how it contributes to soil organic matter (SOM) formation and C storage, under the influence of internal (i.e. compound) and external biotic (i.e. decomposers) and abiotic (i.e. climate) factors, is therefore essential for predicting the future of the terrestrial carbon reservoir.

The cytoplasmic content of cells decreases rapidly during mycelial senescence [63]. Thus, the biochemical composition of the fungal cell wall, where up to 50% of fungal biomass is stored [9], is the main factor regulating the decomposition of fungal necromass. High levels of readily available nitrogen-containing compounds (the majority in proteins, but a minority is also present in chitin [54] and glucans, tend to accelerate the decomposition of fungal necromass [21, 32]. In contrast, the production of melanin, a recalcitrant compound, slows down decomposition and contributes to the formation of SOM and the stabilization of carbon in the soil [43]. However, melanin production is restricted to certain fungal species only [66], protecting their mycelia against harsh environmental conditions [20, 31]. The high abundance of melanized fungi promotes the formation of recalcitrant necromass, thereby enhancing soil carbon sequestration. This is a crucial element in understanding how ecosystems respond to climate change, as a recent study shows that rising temperatures may decrease the share of melanized fungi in tundra biome [48], which may subsequently lead to carbon loss from the soil.

In contrast to fungal residues, which contain structurally complex and chemically resistant polymers such as chitin, glucans, and melanin, bacterial residues are generally considered more labile and less persistent in soil. Bacterial cells lack a thick cell wall rich in recalcitrant compounds, and their necromass typically decomposes faster and contributes less to long-term carbon stabilization [37, 47]. As a result, fungal necromass has received increasing attention in recent years as a key microbial contributor to SOM formation, particularly in cold and nutrient-limited ecosystems such as the Arctic tundra, where long-term inputs to the soil carbon pool are especially important.

Among the external factors that influence necromass decomposition, the composition of the microbial communities that participate in it emerged as a crucial element because microbial taxa differ largely in their saprotrophic capabilities [4, 83]. The initial phase of fungal necromass decomposition, typically spanning the first 40 days (depending on climatic conditions), is dominated by fast-growing opportunistic bacteria and fungi that feed on easily degradable organic compounds. Once these compounds are depleted (> 100 days of decomposition), the opportunists are replaced by oligotrophic bacterial taxa together with saprotrophic and ectomycorrhizal fungi, which possess enzymatic capabilities to degrade more recalcitrant organic compounds [12]. Importantly, very little information is available about microbial communities associated with fungal necromass decomposing for years [41, 42], although some components of the fungal cell wall are not fully decomposed in the soil even after such a long period [80].

Saprotrophic fungi are major decomposers of both plant and fungal necromass due to their ability to produce a wide range of extracellular enzymes that can degrade even recalcitrant material such as plant lignocellulose or fungal melanin [4, 83]. In addition, some ectomycorrhizal fungal species, despite somewhat reduced decomposition capacity, have retained oxidative enzymatic potential from saprotrophic ancestors [35, 38].

Bacteria are also important actors in the decomposition of fungal material. They can rapidly colonize fungal necromass [10, 11] and fungal-derived components [1, 68]. Moreover, it has been suggested that bacterial communities might play an even more important role in fungal necromass decomposition than fungi, especially later in the decomposition process when their abundance increases [39, 70].

Recently, the existence of a core necrobiome, including fungi and bacteria commonly detected on decaying fungal necromass in various ecosystems, has been established [12]. However, it is based on studies where fungal necromass decomposition and succession of associated microbial communities have been studied mainly over short-term periods, averaging 3 months [6, 44], with some longer studies, still not exceeding 2 years [18]. Clearly, fungal necromass, and particularly its melanized form, undergo decomposition processes for many years and its faith in late decomposition stages can significantly affect soil carbon balance. Therefore, understanding long-term development of fungal communities on decomposing mycelia together with identification of potential players in late-stage decomposition can substantially advance our understanding of fungal necromass turnover. Furthermore, despite the pivotal role of fungal necromass in soil carbon sequestration in high-latitude ecosystems, there is a lack of information on fungal necromass decomposition in Arctic tundra.

Hence, in this study, we aimed to determine the loss of fungal necromass of different qualities, as well as the succession of associated fungal and bacterial decomposer communities, over a long decomposition period in the Artic tundra. To this purpose, we incubated mesh bags containing necromass from fungi with low or high C:N and melanin contents in the polar region of the Svalbard archipelago. Over the 3 years of the experiments, we quantified the necromass decomposition and the changes in chemical properties along with the analysis of the diversity and composition of fungal and bacterial communities.

## Materials and methods

### Fungal necromass production

Two fungal species, *Laccaria laccata*, and *Phialocephala fortinii*, were selected to produce high and low-quality necromass. While the isolate of *P. fortinii* was isolated from roots of *Salix sp*. collected on the experimental site, *L. laccata* was obtained from the fungal culture collection of the Institute of Botany of the Czech Academy of Science. Both fungal species were repeatedly reported from tundra ecosystems [75], occur naturally at the field experimental site and interact with local plants, making the selection relevant to the given ecosystem [73].

*L. laccata* is an ectomycorrhizal fungus that forms a hyaline mycelium rich in nitrogen and was therefore selected to represent a high-quality (low C:N, low melanin content) substrate. On the other hand, *P. fortinii* is an endophytic fungus that forms a mycelium with a large amount of poorly degradable melanin and was thus chosen to represent a low-quality (high melanin, high C:N) substrate (Fig. S1, Table S3).

To produce fungal necromass, each species was first cultivated on Petri dishes containing Modified Melin-Norkrans (MMN) medium [46] in the dark at room temperature ( ∼ 20 °C). Plugs from actively growing culture edges were individually transferred to a liquid medium composed of 20 g L^−1^ of malt extract in distilled water (50 ml of media in 250 ml Erlenmeyer flasks). Inoculated cultures with both fungal species were kept in a dark room at a temperature of 25 °C. Due to the different cultivation requirements of each fungal species, cultures of *P. fortinii* were placed on the shaker, whereas stationary culture was used for *L. laccata*. Mycelia were harvested after three weeks, thoroughly washed, freeze-dried, and kept in a freezer (− 20 °C) until further processing. Harvested biomass was also used for chemical analysis and melanin content determination.

Mycobags in the shape of an equilateral triangle (each side 10 cm long) were made of nylon membrane with a mesh of 50 µm, to prevent any plant roots from growing in, but enabling ingrowth of fungal mycelia. One gram of lyophilized mycelium was transferred into each mycobag. In total 116 mycobags were prepared for the field experiment. The prepared mycobags with necromass were subsequently sterilized by two doses of gamma-irradiation D = 500 Gy (Central Bohemian Museum in Roztoky near Prague). The material was transported to the field experiment site under sterile conditions and stored on silica gel to prevent it from getting wet.

### Experimental site description

The experiment was conducted in the polar region of the Svalbard archipelago, specifically at a site in the Billefjorden fjord (78.5304°N, 16.3070°E; 50 m above sea level). The site has previously been used for research and described in published studies [23, 29, 30, 67]. The vegetation cover at the site ranged between 30–50% (on-site observation). Dwarf vegetation typical of the Arctic tundra, such as *Bistorta vivipara*, *Saxifraga oppositifolia*, *Salix polaris*, *Dryas octopetala*, *Silene* sp*.,* and *Cassiope tetragona*, was recorded at the site.

### Design of the experiment and sampling

In July 2019, 10 experimental plots were established on-site at least 10 m apart from each other to capture the heterogeneous environmental conditions as accurately as possible. Twelve mycobags (6 × *L. laccata*, 6 × *P. fortinii*) with necromass were placed in each experimental plot and inserted into the soil at an oblique angle of 45° to a depth of approximately 7 cm. This corresponds to the depth with the highest root density, where the root symbiont biomass naturally occurs. A botanical survey and recording of GPS coordinates were carried out in each experimental plot (Table S2).

The experiment was established on 10 July 2019. A total of 116 mycobags (58 × *P. fortinii*, 58 × *L. laccata*) were placed in the soil. The first sampling was performed after 17 days, on 27 July 2019 (2.5 weeks; T1), and 36 mycobags (18 × *P. fortinii*, 18 × *L. laccata*) were collected. The second sampling was performed after 59 days of incubation on 7 September 2019 (2 months; T2), and 32 mycobags (16 × *P. fortinii*, 16 × *L. laccata*) were collected. The third collection was done after 1122 days (> 3 years; T3) of incubation on 5 August 2022, 47 mycobags were collected (24 × *P. fortinii*, 23 × *L. laccata*), while one *L. laccata* mycobag could not be found (Table S1). The collected material was transported on ice to the laboratory within 72 h and freeze-dried immediately after transport. The samples were stored in a freezer (− 20 °C) until further processing. The collected samples were used to determine substrate loss, chemical composition, and the composition of fungal and bacterial communities associated with decaying fungal necromass.

### Chemical analysis

#### Carbon and nitrogen content

The samples were sent to an external laboratory located at the Institute of Botany of the Czech Academy of Sciences in Průhonice, Czech Republic, for analysis of total carbon (C) and nitrogen (N) content. The analysis of total C content was carried out using the sulphochromic oxidation method, while N content was determined by sulphuric acid mineralization with the addition of selenium and sodium sulphate, followed by the conversion to ammonium ions.

#### Determination of melanin content

To evaluate the melanin content in the initial fungal necromass, a modified quantitative colorimetric assay was used. The assay method was similar to the one described by Fernandez et al. [21]. In this assay, Azure A dye was used as it strongly binds to melanin, which facilitates the measurement of changes in absorbance after the dye solution comes in contact with melanin.

First, a solution of Azure A dye (Thermo Fisher Scientific) was prepared by dissolving the dye in 0.1 M HCl. The solution was filtered through a nitrocellulose membrane with a pore size of 0.45 µm and diluted to an absorbance of 0.665 at a wavelength of 610 nm. Subsequently, a standard absorbance curve was constructed using melanin isolated by acid hydrolysis from lyophilized *P. fortinii* mycelia. The mycelium was placed in 6 M HCl and incubated at 80 °C for four days. The mixture was then filtered through filter paper and the solid component (melanin) was washed several times with distilled water. The isolated melanin was subsequently lyophilized and stored at -20 °C. Melanin was combined with 3 ml of Azure A dye solution at final concentrations of 0.02 mg/ml, 0.04 mg/ml, 0.06 mg/ml, and 0.08 mg/ml. The mixture was incubated at laboratory temperature for 90 min. Subsequently, the mixture was filtered through a syringe tip filter with a pore size of 0.45 µm (Sigma-Aldrich). The absorbance of the filtrates was measured with a spectrophotometer at 610 nm.

### Molecular analysis

#### DNA extraction and PCR amplicon sequencing

Total genomic DNA was extracted from all 115 samples using the modified Miller method [62]. DNA from the first two collection times (2.5 weeks, 2 months) was extracted in duplicates using 50 mg of freeze-dried material. Due to the small amount of material, only one replicate was available for DNA extraction in the third sampling time (3 years). The respective replicates were purified using GeneClean Turbo Kit (MP Biomedicals) and pooled before PCR. Amplification of the fungal ITS2 region, located between the 5.8S and LSU regions of the rDNA, was performed using barcoded gITS7/ITS4 primer pairs [27]. The V4 region of bacterial 16S rDNA was amplified using barcoded 515F/806R primer pairs (Caporaso et al., 2011). PCR was performed in triplicates and negative controls were included in all PCR reactions.

Each PCR reaction contained 5 µL of 5 × buffer for Q5 High-Fidelity DNA polymerase (New England Biolabs, Inc.), 5 µL of 5 × Q5 HighGC Enhancer (New England Biolabs, Inc.), 0.5 µL of 10 mM PCR Nucleotide mix (Bioline), 1.5 µL of 10 mg ml − 1 BSA (GeneON), 0.25 µL of the Q5 High-Fidelity DNA polymerase (New England Biolabs, Inc.), 1 µL of each 10 μM forward and reverse primer (Sigma-Aldrich), 9.75 µL of H2O, and 1 µL of the template DNA. The PCR conditions for the fungal ITS2 region were: initial denaturation for 5 min at 94 °C; 30 cycles of 30 s at 94 °C, 30 s at 56 °C, 30 s at 72 °C; followed by an extension at 72 °C for 7 min. The PCR conditions for the bacterial V4 region were: initial denaturation for 4 min at 94 °C; 25 cycles of 45 s at 94 °C, 60 s at 50 °C, 75 s at 72 °C; followed by an extension at 72 °C for 10 min. Amplicon triplicates were combined, purified using a MinElute Kit (Qiagen), and quantified with a Qubit™ dsDNA BR Assay kit (Thermo Fisher Scientific). Sequencing libraries were prepared using the TruSeq PCR-free Kit (Illumina) and sequenced in-house using Illumina MiSeq (2 × 250).

### Bioinformatic data processing

The fungal (gITS7/ITS4) and bacterial (515F/806R) datasets were analyzed separately using the SEED 2.0 pipeline [74]. Initially, the fastq-join [2] was used to combine the pair-end reads, followed by quality filtering with a mean quality score of 30 as a cutoff. Before further processing, the fungal ITS2 region was extracted using ITSx v1.0.8 [51]. The remaining sequences were clustered at a 97% similarity level using UPARSE implemented within USEARCH [17] after removing chimeric sequences. The most abundant sequence for each operational taxonomic unit (OTU) was selected as the representative sequence, and BLASTn was used for identifications against relevant databases: UNITE version 9 dynamic release [50] for fungi and Ribosomal Database Project [15] for bacteria (both downloaded on 15 March 2023). Non-fungal or non-bacterial sequences were discarded from the datasets accordingly, as well as sequences of the genera *Laccaria* and *Phialocephala* (although they were represented in less than 0.1% of the dataset). Sample A014 (2.5 weeks; low-quality) was removed from the analysis due to a small number of bacterial sequences. The sequence data were deposited in SRA (PRJNA1187830). The taxonomic assignments of the 30 most abundant fungal OTUs were cross-validated by performing BLASTn searches against the GenBank database [64]. In cases where discrepancies arose between the results obtained from the two approaches, preference was given to the taxonomic assignments derived from the GenBank database on the genus level. Subsequently, fungal genera were assigned to putative ecology (primary lifestyle) using FungalTraits [55]. The ecology of some fungi has been changed to the “mold” category according to the available literature [69] and previous experience. The categories ‘litter saprotroph’, ‘soil saprotroph’, ‘wood saprotroph’, ‘dung saprotroph’, and ‘unspecified saprotroph’ have been merged into one category ‘saprotroph’. The functional categories of bacteria were determined according to Trivedi et al. [71], who classified major bacterial groups as copiotrophs or oligotrophs based on their relative associations with labile versus recalcitrant carbon pools and life-history traits such as growth rate and carbon use efficiency.

### Statistical analysis

All statistical analyses and data visualization were performed in R software 4.0.5 [58].

Differences in the chemical composition between fresh *P. fortinii* and *L. laccata* biomass (N content, C:N ratio, melanin content, and melanin:N ratio) were tested by a two-sample t-test.

Changes in substrate chemistry in fungal necromass during the decomposition (C:N ratio) were tested using ANOVA followed by Tukey HSD posthoc test. The response variable was log-transformed if necessary to meet the normal distribution criterion. Incubation time, necromass type, and their interaction were used as explanatory variables.

Linear mixed-effect (LME) models were used to analyze the amount of fungal necromass remaining and changes in fungal and bacterial richness using the „lmer “ function from the „lme4 “ package [5]. Incubation time, necromass type, and their interaction were used as fixed effect predictors. Experimental blocks were included as a random effect.

To calculate the species (OTUs) richness of bacterial and fungal communities, we standardized the data using subsampling. This involved selecting an equal number of sequences from each sample to ensure consistency across samples. Specifically, we used 455 sequences for the bacterial dataset and 835 sequences for the fungal dataset. This approach allowed us to compare species richness among samples without the influence of varying sequence counts in individual samples.

To evaluate the composition of fungal and bacterial communities associated with decaying fungal necromass, we utilized relative abundance, representing the number of sequences normalized by the total number of reads per sample. Fungal and bacterial community compositions were analyzed using nonmetric multidimensional scaling (NMDS) with the „metaMDS” function from the „vegan “ package [52], employing Bray–Curtis distance on Hellinger-transformed OTU matrices. Centroids were calculated and displayed in the NMDS plot for each variant. Permutational multivariate analysis of variance (PERMANOVA) with the „adonis “ function from the „vegan “ package was used to investigate the effect of incubation time, necromass type, and their interaction on the composition of fungal and bacterial communities. Experimental blocks were included as “strata” in the PERMANOVA analysis. The pairwise PERMANOVA with Holm’s P-value correction was used to detect differences between variants.

The „LinDa” package [84] was used to perform a differential relative abundance test. The statistical model included two fixed effects representing the necromass type and the time, and one random effect representing the blocks. This analysis returned two parameters that were used to estimate whether there was a difference in relative abundance between taxa. These parameters are fold change (FC) and adjusted p-value, due to the multiple tests involved. Log2(FC) was computed and represents the number of times the relative abundance increases/decreases concerning a reference level. Reference levels were set for both necromass type, i.e. high-quality (*L. laccata*), and for time, i.e. 2 months (T2).

## Results

### Fungal necromass loss

**Fig. 1:**
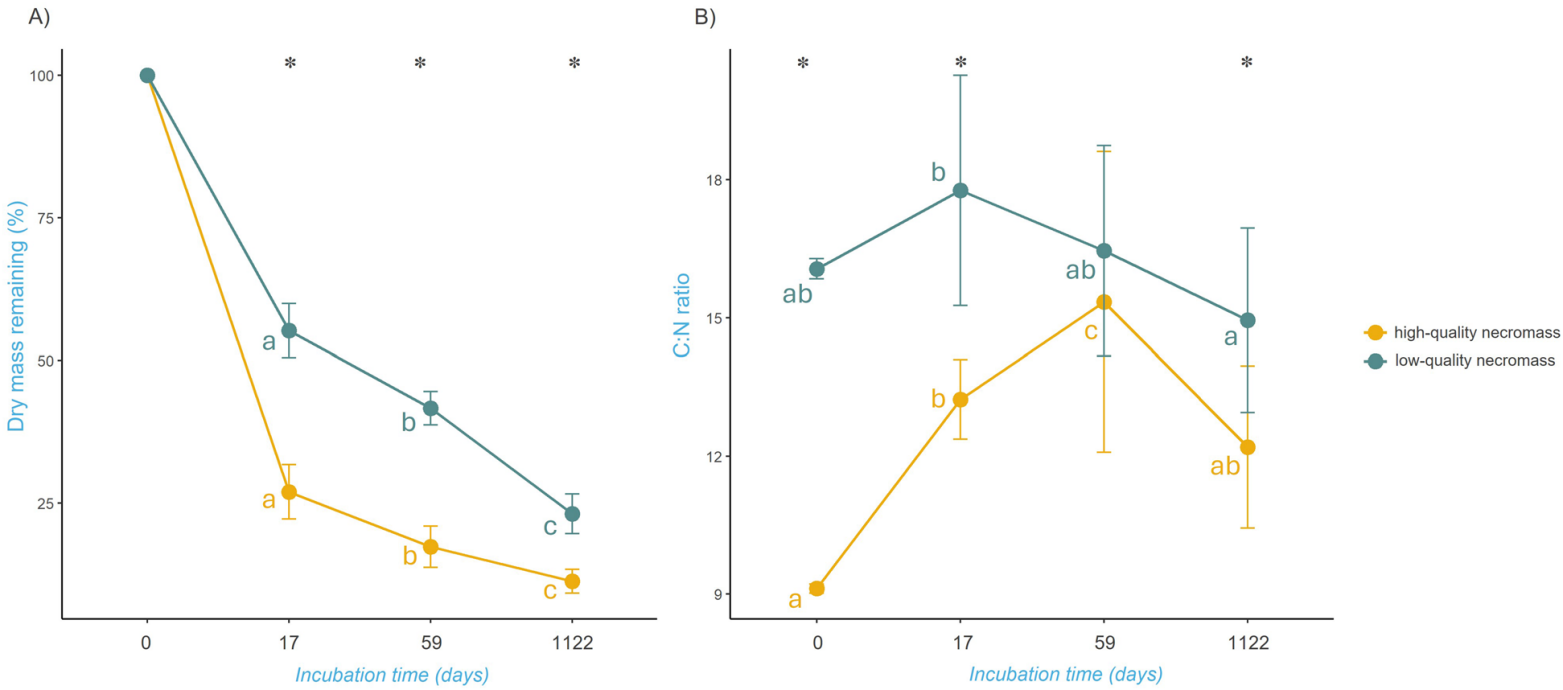
Dry mass loss (**A**) and chemical changes (**B**) of decomposing fungal necromass. **A** Mean percent remaining mass (± SD) of high-quality (yellow) and low-quality (blue) fungal necromass after 17 days), 59 days, and 1122 days of incubation. **B** Mean C:N ratio (± SD) of high-quality (yellow) and low-quality (blue) fungal necromass of initial material (T0) and changes during decomposition after 17 days, 59 days and 1122 days of incubation. Letters indicate significant differences between time points and asterisks indicate significant differences between necromass types tested (linear mixed effect model followed by Tukey HSD posthoc test with Bonferroni P-value correction)

The loss of fungal necromass was significantly affected by incubation time, necromass type, and their interaction (*p* < 0.001, Conditional R^2^ = 0.95; Table S4). The *P. fortinii* low-quality necromass mass loss was slower than the *L. laccata* high-quality necromass. In general, there was a rapid loss of *L. laccata* necromass (high-quality) during the initial phase of incubation (2.5 weeks), with an average of 73.0 ± 4.76% being lost. In comparison, *P. fortinii* necromass (low-quality) experienced a slower mass loss of 45.0% ± 4.76% during the same period. By the end of the later phase of incubation (2 months), 83 ± 3.63% of *L. laccata* necromass and 58 ± 4.76% of *P. fortinii* necromass were lost. After 3 years of incubation, the mass of the bags was 11 ± 2.06% and 23 ± 3.46% for *L. laccata* necromass and *P. fortinii* necromass, respectively (Fig. 1A).

### Changes in chemical properties of decomposing fungal necromass

During the incubation period, changes in the chemical composition of necromass were observed in both types of mycelia. The C:N ratio in fungal necromass was significantly affected by incubation time, necromass type, and their interaction during decomposition (*p* < 0.001, AdjR^2^ = 0.46; Fig. 1B, Table S5). Regarding the *L. laccata* high-quality necromass, we noted a rise C:N ratio after 2.5 weeks of incubation, followed by a subsequent increase after 2 months of incubation. However, after 3 years of incubation, a notable decline was observed. The *P. fortinii* low-quality necromass exhibited a distinct pattern. After 2.5 weeks, a slight increase was observed, followed by a continuous decline after 2 months and continuing after 3 years, with necromass at 2.5 weeks being significantly different from that at 3 years, while the initial time point and the 2-month mark did not show significant differences from the other time points.

### Metabarcoding of necromass-associated microbial communities

After removing singletons, chimeras, and non-fungal sequences, the fungal dataset consisted of 1,562,171 sequences. These sequences were clustered into 1,096 fungal operational taxonomic units (OTUs). The sequencing data revealed that the communities associated with decaying fungal necromass were primarily composed of Ascomycota (719 OTUs; 61% sequences), followed by Mortierellomycota (38 OTUs; 21% sequences), Mucoromycota (16 OTUs; 12% sequences), and Basidiomycota (320 OTUs; 6% sequences).

The bacterial dataset comprised 1,891,684 sequences after the removal of singletons, chimeras, and non-bacterial sequences. These sequences were clustered into 3,503 bacterial OTUs. The decaying fungal necromass was predominantly occupied by Proteobacteria (1,387 OTUs; 54% sequences), followed by Bacteroidetes (367 OTUs; 27% sequences) and Actinobacteria (312 OTUs; 10% sequences).

### Fungal and bacterial richness on decomposing fungal necromass

**Fig. 2:**
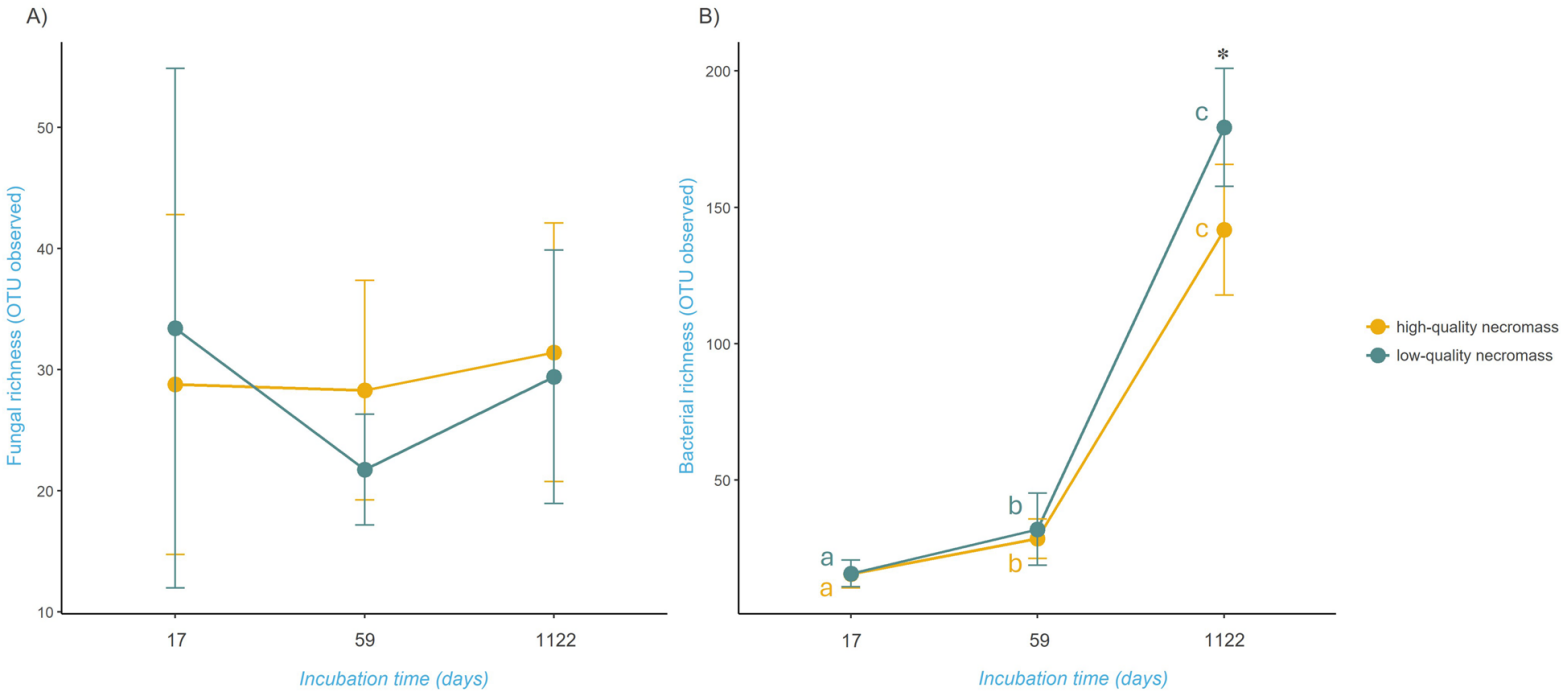
Fungal (**A**) and bacterial (**B**) richness on decomposing fungal necromass. Mean percentage of fungal (**A**) and bacterial (**B**) richness (± SD) associated with high-quality (yellow) and low-quality (blue) fungal necromass after 17 days, 59 days, and 1122 days of incubation. Letters indicate significant differences between time points and asterisks indicate significant differences between necromass types tested (linear mixed effect model followed by Tukey HSD posthoc test with Bonferroni P-value correction)

No significant effect of incubation time, necromass type, or their interaction on fungal richness was found (Fig. 2A, Table S6). On the other hand, both incubation time and type of necromass had a significant effect on the richness of bacteria on fungal necromass, but not their interaction (*p* < 0.001, Conditional R^2^ = 0.95; Fig. 2B, Table S7). Bacterial richness increased with incubation time, with significantly higher bacterial richness on *P. fortinii* necromass than on *L. laccata* necromass after 3 years.

### Fungal community associated with decomposing fungal necromass

**Fig. 3:**
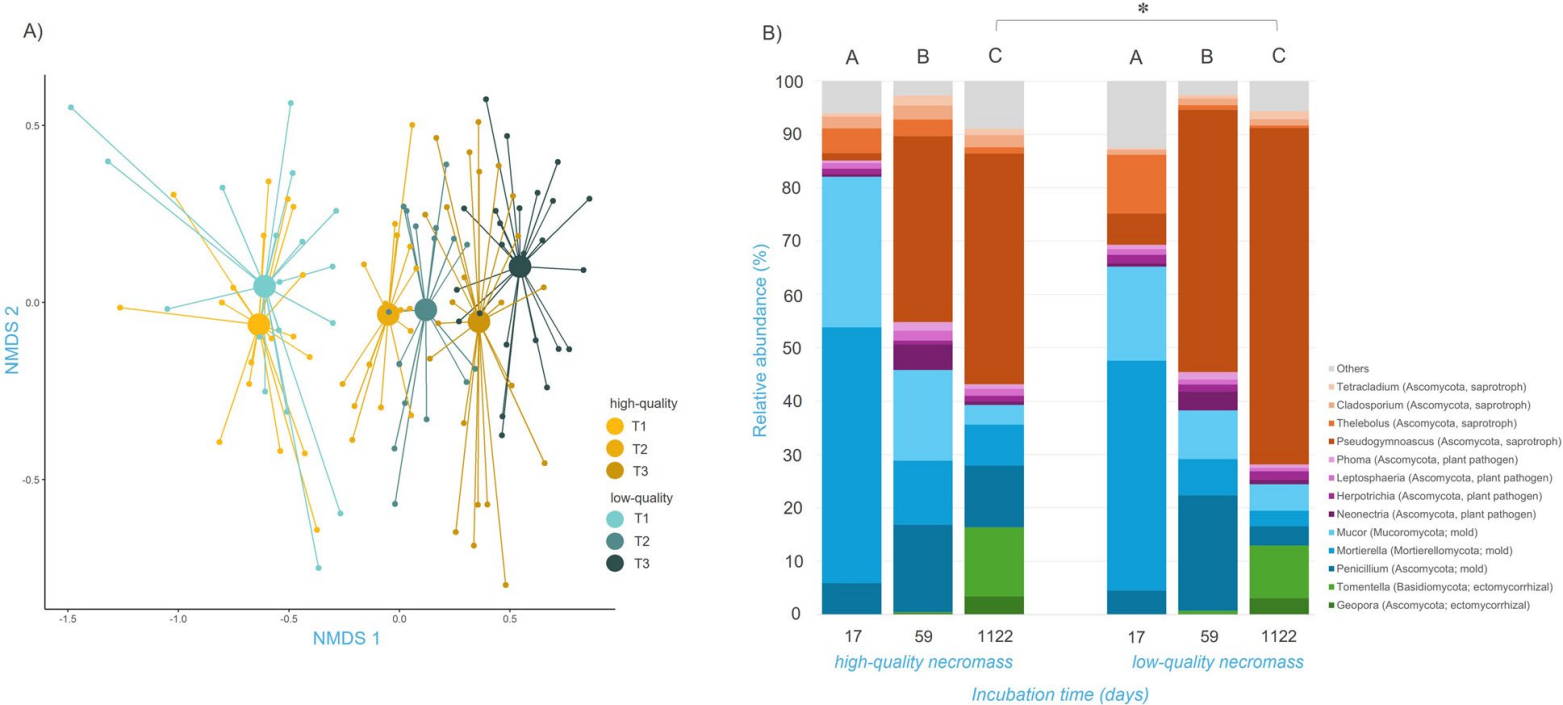
Changes in fungal community composition associated with decaying fungal necromass. **A** NMDS analysis of fungal communities colonizing fungal necromass depending on incubation time and necromass quality with a stress value of 0.199. Small circles represent individual samples (n = 115) and large circles represent centroids. Shades of yellow indicate community succession on high-quality necromass and shades of blue indicate succession on low-quality necromass. **B** Relative abundance of the most represented fungal genera colonizing the decaying fungal necromass depending on incubation time and necromass type. The selected colors refer to the affiliation to the functional group. Letters indicate significant differences between time points and asterisks between necromass types tested (pairwise PERMANOVA with Holm P-value correction)

NMDS ordination showed that the overall fungal community associated with decomposing fungal necromass was structured mainly by incubation time and less so by necromass type. Congruently, PERMANOVA revealed a predominant effect of incubation time (*p* < 0.001, AdjR^2^ = 0.22) on the composition of fungal communities associated with decomposing necromass, over the necromass type (*p* < 0.01, AdjR^2^ = 0.01; Fig. 3A, Table S8). Although the overall effect of the necromass type on the composition of fungal communities was rather small, we identified a significant difference in the composition of fungal communities associated with decomposing necromass after three years of incubation, using pairwise PERMANOVA analysis (Fig. 3B).

The initial phase of decomposition was characterized by a predominance of fast-growing molds, mainly represented by the genera *Penicillium* (Ascomycota), *Mortierella* (Mortierellomycota), and *Mucor* (Mucoromycota). In the later phase of decomposition, the decrease in the proportion of molds was compensated by the increase of saprotrophic fungi, especially of the genus *Pseudogymnoascus* (Ascomycota). Finally, after three years of incubation, the fungal community was especially rich in the genus *Pseudogymnoascus*, but we also observed an increase in the relative abundance of ectomycorrhizal fungi, represented mainly by the genera *Tomentella* (Basidiomycota) and *Geopora* (Ascomycota). Significant differences in the composition of fungal communities between necromass types after three years of incubation were due to a higher share of molds and ectomycorrhizal fungi in the high-quality necromass, compared to higher abundance of saprotrophs in the low-quality necromass (Fig S2A, Fig S3a, Fig S3b).

### Bacterial community associated with decomposing fungal necromass

**Fig. 4:**
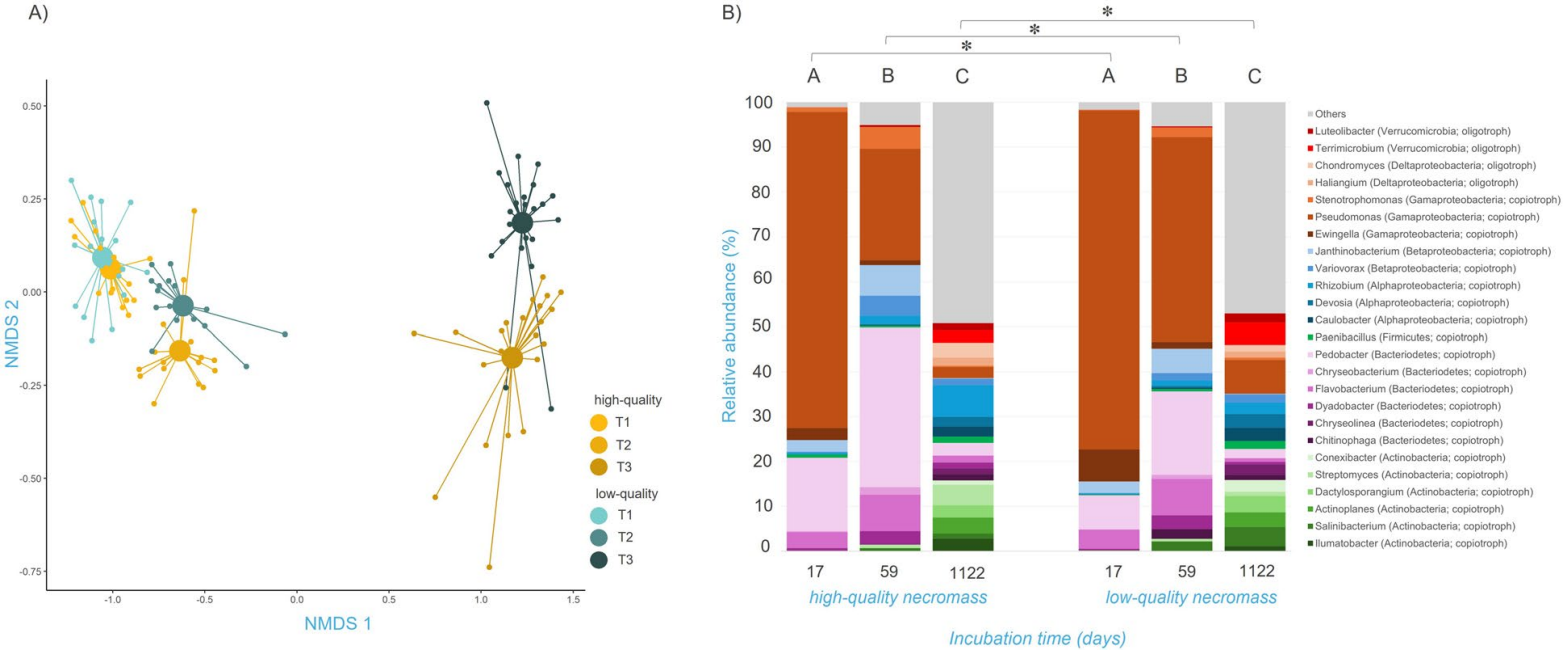
Changes in bacterial community composition associated with decaying fungal necromass. **A** NMDS analysis of bacterial communities colonizing fungal necromass depending on incubation time and necromass quality with a stress value of 0.0516. Small circles represent individual samples (n = 114) and large circles represent centroids. Shades of yellow indicate community succession on high-quality necromass and shades of blue indicate succession on low-quality necromass. **B** Relative abundance of the most represented bacterial genera colonizing the decaying fungal necromass depending on incubation time and necromass type. The selected colors refer to the affiliation to bacterial phylla. Letters indicate significant differences between time points and asterisks between necromass types tested (pairwise PERMANOVA with Holm *P*-value correction)

The NMDS ordination revealed that the bacterial community associated with decomposing fungal necromass was primarily influenced by the time of incubation, with a lesser impact of the type of necromass and their interaction (Fig. 4A). PERMANOVA analysis confirmed that the incubation time had a predominant effect (*p* < 0.001, AdjR2 = 0.60) on the composition of bacterial communities associated with decomposing necromass, while the influence of necromass type (*p* < 0.01, AdjR2 = 0.01) and their interaction (*p* < 0.01, AdjR2 = 0.01) was comparatively minor (Table S8). However, pairwise PERMANOVA analysis indicated a significant difference in bacterial community composition between biomass types at each sampling point (Fig. 4B).

The bacterial community associated with the decaying fungal necromass was characterized by a high relative abundance of *Pseudomonas* (Proteobacteria) and *Pedobacter* (Bacteroidetes) in the initial phase. In the later phase, there was a decrease in the relative abundance of *Pseudomonas* and an increase in *Pedobacter* and *Flavobacterium* (Bacteroidetes). After three years of incubation, we observed a decrease in the relative abundance of the previously dominant genera, whereas we observed an increase in the relative abundance of various genera of bacteria from the phyla Actinobacteria. At this stage, none of the bacterial genera exceeded 10% in their relative abundance (Fig. 4B). Looking at the functional categories of bacteria, we observed an increase in the relative abundance of oligotrophic bacteria after three years of incubation (Figs. S2B, S3c, S3d). In contrast to the fungal communities, the bacterial communities showed the occurrence of additional taxa after three years of incubation, which also corresponds to a significantly increased bacterial richness.

## Discussion

In this study, we addressed decomposition rate and microbial communities associated with two types of fungal necromass differing in chemical composition in a long-term field experiment in Arctic tundra soil.

### The decomposition and chemical changes of decaying fungal necromass

Our findings indicate that the principles governing fungal necromass decomposition in the Arctic tundra align with those previously observed in other ecosystems [6, 10, 11, 18, 19, 41]. The dynamics of fungal necromass decomposition in arctic tundra soils follow the typical pattern, with a rapid initial phase, possibly explained by abiotic leaching [44] and the attractiveness of an easily decomposable substrate, leaving more recalcitrant substrates, which hamper decomposition in the later decomposition phase [19]. In addition, our results indicate that the chemical composition of the decomposing material might control this process. This was expected as melanin contributes to the recalcitrance of fungal cell walls [18, 19], Fernandez & Koide, 2014) and protects against the direct leaching of soluble materials [44], while nitrogen content has a positive effect on the colonization of dead mycelia and is associated with a higher decomposition rate [6, 11, 18].

In addition to analysis of the chemical composition of the initial necromass, we monitored the changes over time. While the C:N ratio of *P. fortinii* (low-quality necromass) did not show profound changes during the decomposition process, we identified a strong increase in the C:N ratio in the early decomposition phase of *L. laccata* (high-quality necromass). This suggests that the more easily decomposable necromass fraction may serve as an easily available source of N, which is often considered a limiting resource in tundra soils [25, 65]. After three years of incubation, we observed a decrease in the C:N ratio, in the necromass of *L. laccata* particularly, indicating potential accumulation of fresh microbial biomass associated with decomposing mycelia. Despite this observed decrease in the C:N ratio, suggesting microbial activity and biomass accumulation, we found clear residues of the original necromass material in all harvested mycobags, although in this experimental setup, we are not able to quantitatively distinguish what part of the material is original mycelium and what part is newly grown biomass or necromass. Nevertheless, up to 11% of hyaline and 23% of melanized fungal necromass remained even after three years of decomposition. This indicates that even after three years of incubation, complete decomposition of the fungal necromass did not occur, which corresponds to previous findings, that at a later stage of decomposition, when the decomposition rate is close to zero, there is still a relatively large amount of material left in the mycobags [10]. Therefore, we conclude that this poorly decomposable material most likely contributes to the accumulation of SOM and carbon storage in soil and deserves further attention.

While we must acknowledge that our chosen methodology excludes the potential influence of soil invertebrates on fungal necromass decomposition, it is known that the activity of invertebrates in the Arctic tundra is generally low in comparison to other ecosystems [77]. In these environments, fungi and bacteria are recognized as the primary decomposers, playing the most significant roles in the breakdown of organic material [72].

### Necromass-associated microbial communities in Arctic tundra

Although the fungal OTUs richness associated with decaying fungal necromass did not change in time, we observed a succession of fungal communities developing on decaying fungal necromass over three years. Changes in necromass-associated fungal communities were rather related to shifts in the relative abundance of fungal taxa than replacements of early successional species with late successional species, which corresponds to patterns previously documented by other studies [40]. Therefore, the development of fungal communities on decomposing fungal necromass resembles a succession of fungal decomposers associated rather with decaying plant roots [33, 34] than with decaying plant leaves [76] or decomposing wood [57], where fungal communities undergo more profound successional changes associated with fungal species replacement. This indicates potentially similar assembly rules behind the structuring of fungal communities associated with soil-borne biomass, regardless of its origin.

In general, fungal communities associated with decomposing fungal necromass in the Arctic tundra are composed of many species that were repeatedly reported from decaying fungal necromass [12]. In the initial phases, decomposing fungal necromass was mainly associated with *Mortierella*, *Mucor*, and *Penicillium*, which are fast-growing molds and some of the earliest colonizers of fungal necromass [12]. Our study indicates that they can successfully colonize decomposing mycelia, regardless of its quality, within the first two weeks of the decomposition process even in harsh conditions of Arctic tundra. The predominance of saprotrophic fungi from the genus *Pseudogymnoascus* characterized the two later decomposition stages, with profound increase in the latest one. The dominance of *Pseudogymnoascus* was particularly pronounced on low quality necromass after more than three years of decomposition. Although *Pseudogymnoascus* belongs to the core necrobiome [12], it has never been reported as a dominant member of fungal communities developing on decomposing fungal necromass. *Pseudogymnoascus*, which is often found in cold Arctic soils [7, 61], produces various extracellular enzymes including lipases, chitinases, and cellulases, indicating the participation of their producer in the degradation of plant and fungal necromass. *Pseudogymnoascus* is also able to grow on fulvic and humic acids [60]. Our results together with its psychrophilic properties and enzymatic capabilities show the importance of *Pseudogymnoascus* in the decomposition of fungal necromass in the Arctic tundra soils.

Interestingly, we found a relatively low abundance of Basidiomycetes, which is uncommon in fungal necromass-associated communities [10, 11]. Nonetheless, Ascomycetes fungi are dominant in the soil of the Svalbard archipelago where the relative abundance of their sequences can reach up to 80% [78, 82], which indicates strong influence of the composition of local fungal species pool on structuring fungal necromass associated fungal communities.

While only molds and saprotrophic fungi were recorded in fungal communities associated with decomposing fungal necromass during the first six weeks of decomposition, several taxa of ectomycorrhizal fungi appeared later. Ectomycorrhizal fungi have previously been shown to associate with decaying fungal necromass in various ecosystems, under the condition that ectomycorrhizal vegetation is present at the experimental site [6]. Extracellular enzymatic activity of certain ectomycorrhizal fungi enables them to potentially acquire nitrogen present in organic compounds for their host plants [38]. In our field experiment, we observed two genera of ectomycorrhizal fungi associated with late-stage decomposition, namely the genera *Tomentella* and *Geopora*. Both are globally distributed according to the GlobalFungi database [75] and were commonly reported from the Arctic tundra. *Tomentella* has previously been observed as a common associate with decomposing fungal necromass [12]. *Geopora*, on the other hand, has not been reported in any similar study. Although ectomycorrhizal fungi are considered to have very low host specificity, both *Tomentella* and *Geopora* have been observed in the roots of the polar ectomycorrhizal plants *Salix polaris* [22] and *Bistorta vivipara* [49], which were abundant at our experimental site. Considering that necromass C:N was decreasing during the long-term decomposition; it remains highly questionable whether the necromass served as a source of N for the ectomycorrhizal fungi. Further research is needed to understand potential role of fungal necromass as a source of nitrogen for ectomycorrhizal fungi and their hosts.

During our experiment with fungal necromass decomposition, bacterial OTUs richness exhibited a well-known pattern [40] of increase with short incubation time. In forest soils, bacterial diversity on decomposing fungal biomass shows a gradual rise over time, typically after 3–4 weeks to 9–21 weeks of incubation [6, 10, 11]. In contrast, certain ecosystems like savanna grasslands may not demonstrate a similar increase within shorter incubation periods [6]. Long-term studies are limited but have shown relatively stable bacterial OTU richness over extended incubation periods, as observed in boreal forests [44]. However, our study reveals a significant uptick in bacterial richness, observed after more than 3 years of incubation. This increase appears independent of necromass quality and is more pronounced for low-quality residues, suggesting a broader spectrum of bacterial taxa associated with the decomposition of complex necromass. Another possible explanation is that there is not much remaining for bacteria to decompose, and the dominance of primary decomposers has diminished, creating ecological space for a more diverse bacterial community to establish.

Dynamic changes in the richness of bacterial OTUs also reflect the successional development of bacterial communities that are associated with decaying fungal necromass. Among the bacterial genera identified during the early stages of decomposition, particular attention is drawn to *Pseudomonas* (Proteobacteria), which was highly abundant in the early stage of decomposition, and *Pedobacter* (Bacteroidetes), which dominated after six weeks. Notably, both *Pseudomonas* and *Pedobacter* are commonly observed as initial fungal necromass colonizers across various ecosystems [12]. *Pseudomonas* in soil typically adhere to living fungal hyphae [81], and both *Pseudomonas* and *Pedobacter* produce a wide range of extracellular enzymes [28], including chitinases [79], indicating their ability to participate in fungal necromass decomposition.

Bacterial communities associated with fungal necromass are known to be very dynamic over time [12]. Interestingly, in our study, *Pseudomonas* was consistently more abundant on low-quality necromass throughout the incubation period, as were *Streptomyces* (Actinobacteria) and *Caulobacter* (Proteobacteria), suggesting their potential specialization in the degradation of recalcitrant material in Arctic tundra soils and their potential role in soil carbon stabilization. In fact, *Caulobacter* is present in a variety of habitats and known for its ability to thrive in low nutrient conditions and grow in cold temperatures [3], while members of the genus *Streptomyces* are common soil bacteria that are involved in the catabolism of complex molecules and substances such as chitin, xylan, cellulose, and lignin, thus being potentially important in the decomposition of SOM [8, 45]. In addition, they can produce bioactive substances, such as antibiotics, which can limit the growth of other microorganisms and thus maintain their dominance on the decaying material [53].

The observed increase in the relative abundance of oligotrophic bacteria during the third year of incubation is notable, suggesting a succession from copiotrophic to oligotrophic bacteria, consistent with patterns seen in other ecosystems [6, 42], which underscores their role in nutrient cycling and soil carbon storage, particularly in nutrient-limited environments like the Arctic tundra.

## Conclusion

Our study demonstrates that fungal necromass decomposition in Arctic tundra exhibits an initial rapid phase of decay followed by a much slower, recalcitrant phase. This trend aligns with decomposition dynamics observed in other ecosystems. Notably, even after three years of incubation, we identified significant remnants of fungal necromass, indicating that the decomposition process remained incomplete. Although our experimental design does not allow us to differentiate between original necromass and newly formed microbial biomass, these findings suggest that fungal necromass might play a significant role in the long-term sequestration of soil organic matter and carbon in Arctic soils.

Furthermore, we observed contrasting patterns in the development of bacterial and fungal communities associated with the decomposing necromass. While fungal community richness remained relatively stable over time, bacterial richness increased after three years, particularly on low-quality necromass. Fungal communities showed changes in relative abundance, with early colonizing molds giving way to saprotrophic species such as *Pseudogymnoascus*, which completely dominated after three years of incubation. In contrast, bacterial communities showed a more dynamic successional pattern, with early dominance by copiotrophic taxa transitioning to oligotrophic species as decomposition progressed. These findings underscore the contrasting stability of fungal and bacterial communities during long-term decomposition, highlighting the complex interplay between microbial decomposers in Arctic tundra soils. 

## Supplementary Information

Additional file1

Additional file2

## Data Availability

The sequence data were deposited in SRA (PRJNA1187830).
