# Supplementary material for: Contrasting stability of fungal and bacterial communities during long-term decomposition of fungal necromass in Arctic tundra
Source: Environ Microbiome. 2025 Jun 20;20:75. doi: 10.1186/s40793-025-00730-5 (PMC12180224; doi:10.1186/s40793-025-00730-5)

**Contrasting stability of fungal and bacterial communities during long-term decomposition of fungal necromass in Arctic tundra**

Supplementary material

Tables S1 – S8

Figures S1 – S3

**Table S1**. Overview of sampling points and necromass collection during the study.

| Sampling point | Date (dd/mm/yyyy) | Mycobags collected | Necromass type |
| --- | --- | --- | --- |
| T1 (17 days) | 7/27/2019 | 18 | High-quality *(L. laccata)* |
|  |  | 18 | Low-quality *(P. fortinii)* |
| T2 (59 days) | 9/7/2019 | 16 | High-quality *(L. laccata)* |
|  |  | 16 | Low-quality *(P. fortinii)* |
| T3 (1122 days) | 8/5/2022 | 23 | High-quality *(L. laccata)* |
|  |  | 24 | Low-quality *(P. fortinii)* |

**Table S2**. Coordinates and vegetation surveys of sampling blocks.

| Block | Latitude | Longitude | Vegetation survey |
| --- | --- | --- | --- |
| block01 | 78.71268 | 16.4324 | *Bistorta vivipara, Saxifraga oppositifolia, Salix polaris, Oxyria digyna, Carex rupestris, Eutrema edwardsii, Minuartia stricta* |
| block02 | 78.71278 | 16.4361 | *Cerastium arcticum, Saxifraga oppositifolia, Salix polaris, Oxyria digyna, Silene acaulis, Minuartia stricta* |
| block03 | 78.7128 | 16.4326 | *Saxifraga oppositifolia, Salix polaris, Bistorta vivipara, Dryas octopetala, Carex rupestris, Silene apetala, Equisetum variegatum, Silene acaulis* |
| block04 | 78.71282 | 16.43295 | *Bistorta vivipara, Juncus cf. biglumis, Silene acaulis, Oxyria digyna, Saxifraga oppositifolia, Salix polaris* |
| block05 | 78.71285 | 16.4326 | *Bistorta vivipara, Salix polaris, Silene acaulis, Saxifraga oppositifolia, Juncus cf. biglumis, Luzula confusa* |
| block06 | 78.71285 | 16.4335 | *Bistorta vivipara, Minuartia stricta, Salix polaris, Equisetum variegatum, Silene apetala, Carex rupestris, Silene acaulis* |
| block07 | 78.71288 | 16.4345 | *Bistorta vivipara, Salix polaris, Saxifraga oppositifolia, Minuartia stricta, Draba sp., Sagina nivalis, Juncus cf. biglumis, Dryas octopetala, Luzula confusa* |
| block08 | 78.7129 | 16.4357 | *Equisetum variegatum, Oxyria digyna, Luzula confusa, Cassiope tetragona, Luzula confusa, Saxifraga oppositifolia, Dryas octopetala, Cerastium arcticum, Silene acaulis, Salix polaris, Bistorta vivipara* |
| block09 | 78.712945 | 16.4334 | *Salix polaris, Bistorta vivipara, Saxifraga oppositifolia, Juncus cf. biglumis, Carex rupestris* |
| block10 | 78.71295 | 16.4324 | *Bistorta vivipara, Salix polaris, Oxyria digyna, Carex rupestris, Eutrema edwardsii, Silene acaulis, Luzula confusa* |

**Table S3**. Average values and standard deviations for decomposition metrics and chemical properties of fungal necromass over time.

|  |  | Necromass type | | | | | | | | |
| --- | --- | --- | --- | --- | --- | --- | --- | --- | --- | --- |
|  |  | *High-quality necromass (L. laccata)* | | | | *Low-quality necromass (P. fortinii)* | | | | |
|  |  | **T0** | **17 days** | **59 days** | **1122 days** | **T0** | **17 days** | **59 days** | **1122 days** |  |
| Dry mass remaining (%) | | 100 | 26.99 ± 4.76 | 17.33 ± 3.63 | 11.30 ± 2.06 | 100 | 55.21 ± 4.76 | 41.65 ± 2.94 | 23.12 ± 3.46 |  |
| Chemistry | C content (%) | 42.24 ± 2.20 | 48.90 ± 1.04 | 47.53 ± 2.76 | 43.71 ± 5.57 | 50.16 ± 5.01 | 51.32 ±3.40 | 48.83 ± 3.33 | 46.39 ± 1.63 |  |
|  | N content (%) | 4.63 ± 0.29 | 3.71 ± 0.26 | 3.21 ± 0.64 | 3.64 ± 0.61 | 3.13 ± 0.35 | 2.94 ± 0.43 | 3.02 ± 0.43 | 3.16 ± 0.44 |  |
|  | C:N ratio | 9.12 ± 0.10 | 13.23 ± 0.86 | 15.34 ± 3.26 | 12.19 ± 1.76 | 16.06 ± 0.22 | 17.77 ± 2.50 | 16.46 ± 2.28 | 14.95 ± 2.00 |  |
|  | Melanin (mg/g) | 59.58 ± 17.08 |  |  |  | 130.45 ± 9.60 |  |  |  |  |
|  | Melanin:N ratio | 12.98 ± 4.28 |  |  |  | 41.87 ± 2.32 |  |  |  |  |

**Table S4**. Results of the Linear Mixed-Effects Model (LMER) for dry mass remaining. The table summarizes the estimates, confidence intervals (CI), and p-values for the predictors in the LMER model, with dry mass remaining as the dependent variable. The fixed effects include incubation time, necromass type, and their interaction. The model indicates significant negative effects of time on dry mass remaining at 59 days and 1122 days compared to the intercept (baseline at 17 days), and a significant positive effect for low-quality necromass type. The interaction terms suggest that low-quality necromass degrades differently over time, with a less pronounced decrease in mass at 59 days and a more substantial decrease at 1122 days. Random effects are accounted for by the variable "blocks," with a variance (τ00) of 1.28. The model includes 10 blocks with 115 observations, achieving a marginal R² of 0.943 and a conditional R² of 0.948, indicating a high level of explanatory power.

| Predictors | *Estimates* | *CI* | *P-value* |
| --- | --- | --- | --- |
| *(Intercept)* | 27.08 | 25.29 – 28.86 | **0.001** |
| Time [59 days] | -9.81 | -12.21 – -7.41 | **0.001** |
| Time [1122 days] | -15.86 | -18.07 – -13.66 | **0.001** |
| Necromass type [low-quality] | 28.22 | 25.91 – 30.53 | **0.001** |
| Time [59 days] × Necromass type [low-quality] | -3.9 | -7.27 – -0.53 | **0.024** |
| Time [1122 days] × Necromass type [low-quality] | -16.34 | -19.41 – -13.26 | **0.001** |
|  |  |  |  |
| Random Effects |  |  |  |
| σ^2^ | 12.24 |  |  |
| τ_00_ _blocks_ | 1.28 |  |  |
| N _blocks_ | 10 |  |  |
| Observations | 115 |  |  |
| Marginal R^2^ / Conditional R^2^ | **0.943 / 0.948** |  |  |

**Table S5**. Results of the ANOVA for the C:N ratio. This table summarizes the analysis of variance (ANOVA) results for the C:N ratio as the dependent variable. The analysis assesses the effects of necromass type, incubation time, and their interaction on the C:N ratio. The model shows significant main effects of both necromass type (df = 1, F = 61, p = 0.001) and incubation time (df = 3, F = 11.353, p = 0.001), indicating that both factors significantly influence the C:N ratio. Additionally, the interaction between incubation time and necromass type is significant (df = 3, F = 5.364, p = 0.01), suggesting that the effect of incubation time on the C:N ratio varies depending on the necromass type. The residuals have 113 degrees of freedom, and the adjusted R-squared value of 0.4646 indicates that the model explains approximately 46.46% of the variance in the C:N ratio.

| Predictors | df | F.Model | *P-value* |
| --- | --- | --- | --- |
| Necromass type | 1 | 61 | **0.001** |
| Incubation time | 3 | 11.353 | **0.001** |
| Incubation time × Necromass type | 3 | 5.364 | **0.01** |
| Residuals | 113 |  |  |
| Adjusted R-squared | 0.4646 |  |  |

**Table S6**. Results of the Linear Mixed-Effects Model (LMER) for fungal richness (log-transformed). The table presents the estimates, confidence intervals (CI), and p-values for the predictors in the LMER model with fungal richness as the dependent variable. Fixed effects include incubation time, necromass type, and their interaction. The intercept is significant, suggesting a baseline log-transformed fungal richness value of 3.27. Time [59 days] and time [1122 days] show non-significant estimates, indicating no clear effect of time on fungal richness. Necromass type [low-quality] also shows a non-significant effect. Random effects were modeled with "blocks," showing a variance (τ00) of 0.00. The model included 10 blocks with 115 observations, and both marginal and conditional R² were 0.080, indicating low explanatory power of the fixed and random effects.

| Predictors | *Estimates* | *CI* | *P-value* |
| --- | --- | --- | --- |
| *(Intercept)* | 3.27 | 3.10 – 3.44 | **0.001** |
| Time [59 days] | 0.04 | -0.21 – 0.28 | 0.753 |
| Time [1122 days] | 0.13 | -0.09 – 0.35 | 0.256 |
| Necromass type [low-quality] | 0.09 | -0.15 – 0.33 | 0.449 |
| Time [59 days] × Necromass type [low-quality] | -0.34 | -0.69 – 0.00 | 0.052 |
| Time [1122 days] × Necromass type [low-quality] | -0.16 | -0.48 – 0.15 | 0.313 |
|  |  |  |  |
| Random Effects |  |  |  |
| σ^2^ | 0.13 |  |  |
| τ_00_ _blocks_ | 0.00 |  |  |
| N _blocks_ | 10 |  |  |
| Observations | 115 |  |  |
| Marginal R^2^ / Conditional R^2^ | 0.080 / 0.080 |  |  |

**Table S7**. Results of the Linear Mixed-Effects Model (LMER) for bacterial richness (log-transformed). The table displays the fixed effects' estimates, confidence intervals (CI), and p-values for bacterial richness as the dependent variable. The model includes incubation time, necromass type, and their interaction as predictors, with "blocks" as a random effect. The intercept is significant, indicating a baseline log-transformed bacterial richness of 2.71. Time [59 days] and time [1122 days] show significant positive estimates, suggesting that bacterial richness increases over time. The necromass type [low-quality] is not significant, indicating no substantial effect. The interaction term for time [1122 days] × necromass type [low-quality] is significant (p = 0.031), suggesting a notable interaction effect at this time point. Random effects analysis showed a variance (τ00) of 0.01 for blocks. The model used 10 blocks with 114 observations. The marginal R² of 0.942 and conditional R² of 0.951 indicate that the model explains a substantial portion of the variance in bacterial richness.

| Predictors | *Estimates* | *CI* | *P-value* |
| --- | --- | --- | --- |
| *(Intercept)* | 2.71 | 2.58 – 2.84 | **0.001** |
| Time [59 days] | 0.58 | 0.42 – 0.75 | **0.001** |
| Time [1122 days] | 2.22 | 2.07 – 2.37 | **0.001** |
| Necromass type [low-quality] | 0.02 | -0.14 – 0.18 | 0.842 |
| Time [59 days] × Necromass type [low-quality] | 0.07 | -0.16 – 0.30 | 0.544 |
| Time [1122 days] × Necromass type [low-quality] | 0.23 | 0.02 – 0.44 | **0.031** |
|  |  |  |  |
| Random Effects |  |  |  |
| σ^2^ | 0.06 |  |  |
| τ_00_ _blocks_ | 0.01 |  |  |
| N _blocks_ | 10 |  |  |
| Observations | 114 |  |  |
| Marginal R^2^ / Conditional R^2^ | **0.942 / 0.951** |  |  |

**Table S8**. Results of PERMANOVA analyses for the effects of incubation time, necromass type, and their interaction on fungal and bacterial community composition associated with decomposing necromass. This table presents the results of PERMANOVA analyses evaluating the influence of incubation time (17 days, 59 days, and 1122 days), necromass type (high-quality necromass from *L. laccata* and low-quality necromass from *P. fortinii*), and their interaction on microbial community composition. Fungal communities: The results indicate a significant effect of incubation time (df = 2, F = 17.5351, adjusted R² = 0.22098, p = 0.001), demonstrating that fungal community composition varies notably over time. The necromass type also shows a significant effect (df = 1, F = 3.4346, adjusted R² = 0.01433, p = 0.003), though with a smaller effect size. However, the interaction between incubation time and necromass type is not significant (df = 2, F = 0.9757, adjusted R² = 0, p = 0.304), suggesting that the combined effect of these factors does not influence the fungal community composition. Bacterial communities: The incubation time has a strong and significant effect on bacterial community composition (df = 2, F = 95.474, adjusted R² = 0.59955, p = 0.001), indicating major shifts in bacterial community structure over time. The necromass type also significantly affects bacterial communities, albeit with a smaller effect size (df = 1, F = 7.128, adjusted R² = 0.01392, p = 0.002). The interaction between incubation time and necromass type is significant (df = 2, F = 4.343, adjusted R² = 0.01007, p = 0.002), showing that the effect of incubation time on bacterial community composition differs depending on the type of necromass.

| Microbial communities | Variable | df | F.Model | R^2^ _adj_ | *P-value* |
| --- | --- | --- | --- | --- | --- |
| Fungal communities | Incubation time | 2 | 17.5351 | **0.22098** | **0.001** |
|  | Necromass type | 1 | 3.4346 | **0.01433** | **0.003** |
|  | Incubation time × Necromass type | 2 | 0.9757 | 0 | 0.304 |
| Bacterial communities | Incubation time | 2 | 95.474 | **0.59955** | **0.001** |
|  | Necromass type | 1 | 7.128 | **0.01392** | **0.002** |
|  | Incubation time × Necromass type | 2 | 4.343 | **0.01007** | **0.002** |

**Fig. S1**. Initial material chemistry

Mean (±SD) of (A) nitrogen content (%), (B) melanin content (mg/g), (C) C:N ratio, and (D) melanin:N ratio of high-quality (yellow) and low-quality (blue) fungal necromass (n=3).


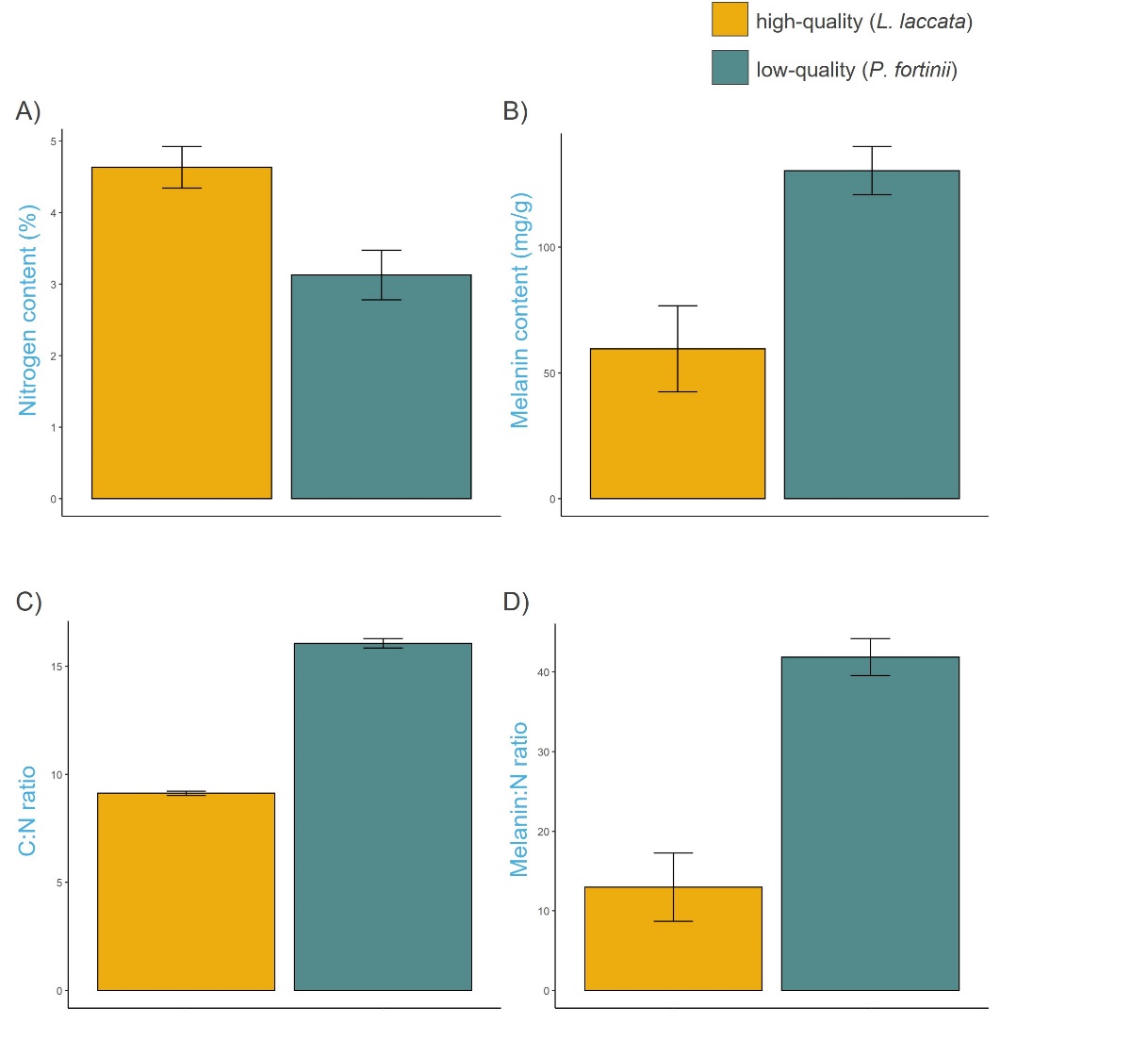


**Fig. S2**. Relative abundance of different functional groups of fungi (A) and bacteria (B) colonizing fungal necromass depending on incubation time and type of necromass.


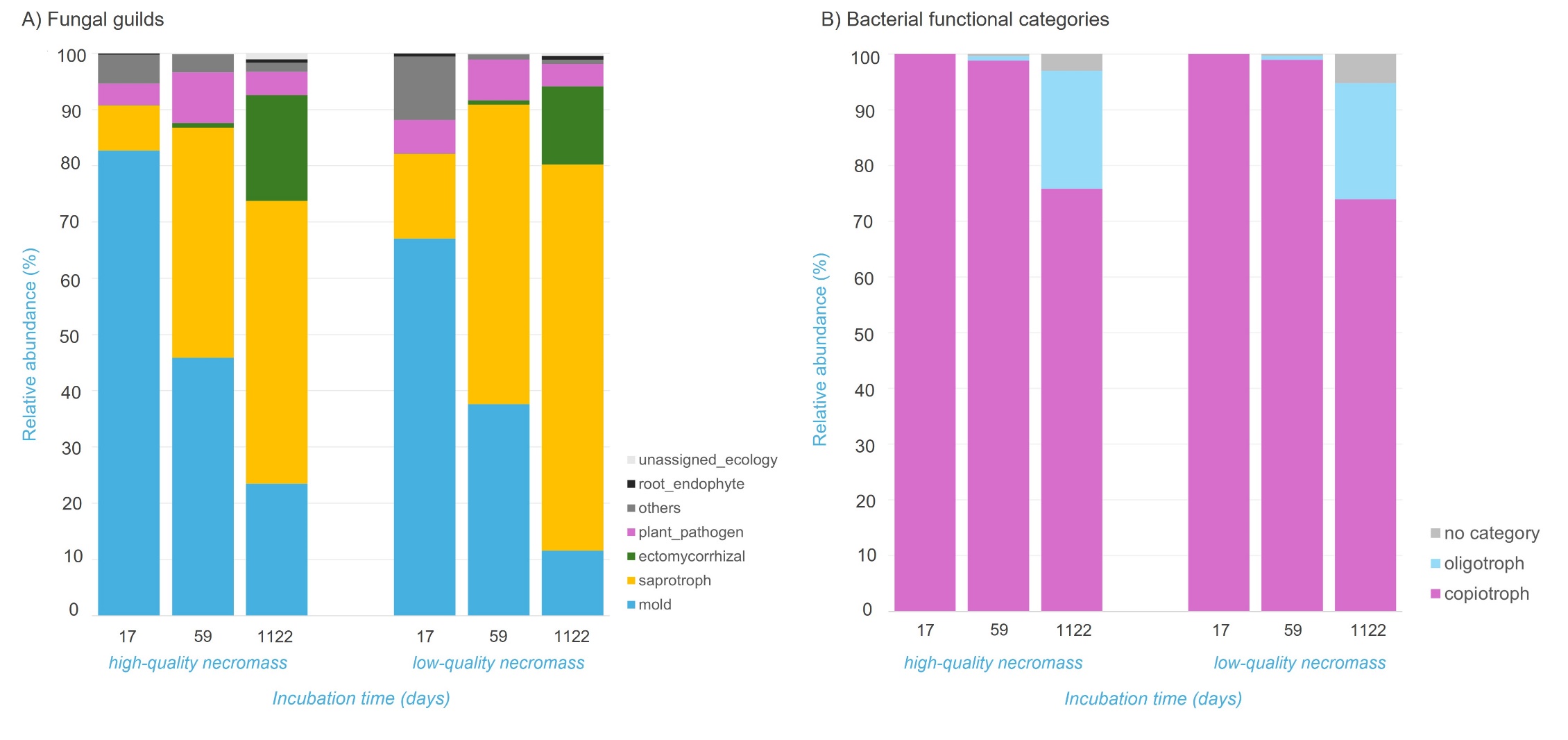


**Fig. S3**. Differential analysis of the effect of necromass quality type (high- vs. low-quality) and incubation time (T1 vs. T2, and T2 vs. T3), on necromass associated (a) fungal genera, (b) fungal phyla, (c) bacterial genera, and (d) bacterial phyla. Circles are colored based on (a) fungal ecology assignment and (c) bacterial functional group assignment and the size of the circle represents the average transformed relative abundance (relative abundance/10). All fungal phyla and genera, all bacterial phyla but only genera that were represented in at least 0.1% of the entire dataset were included in the analysis. Only significant results are assigned a name.


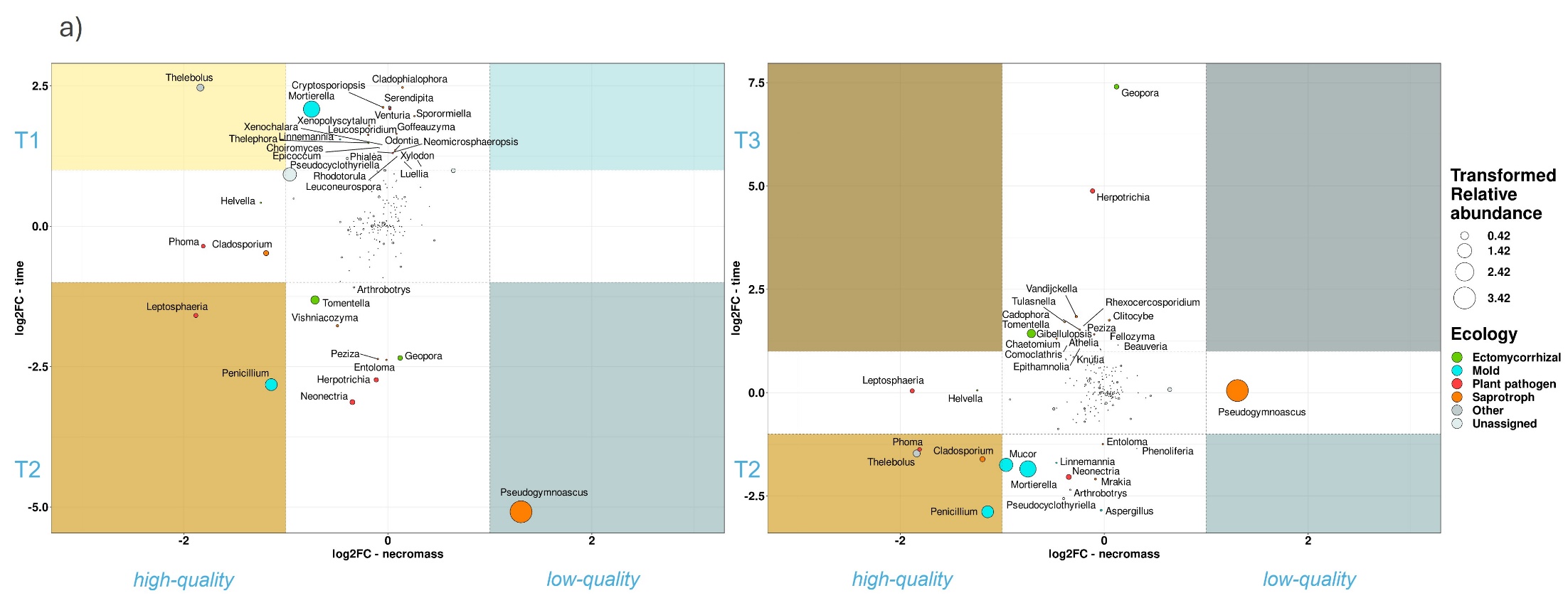


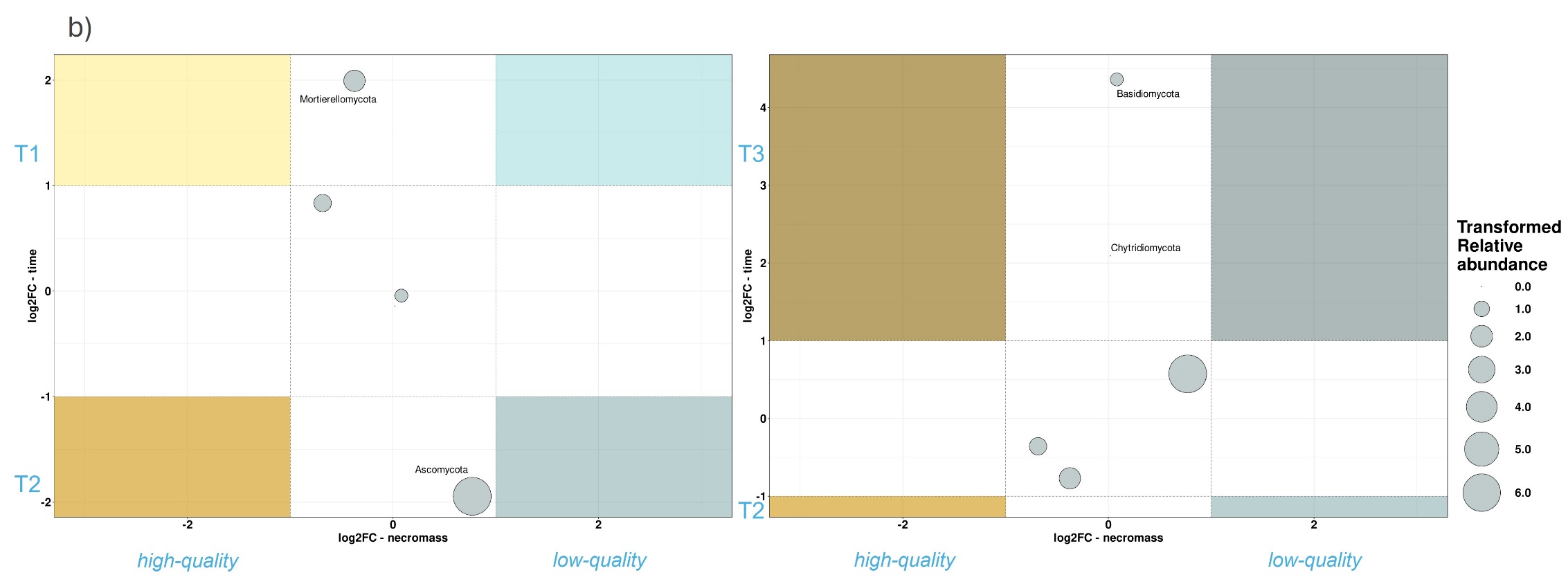


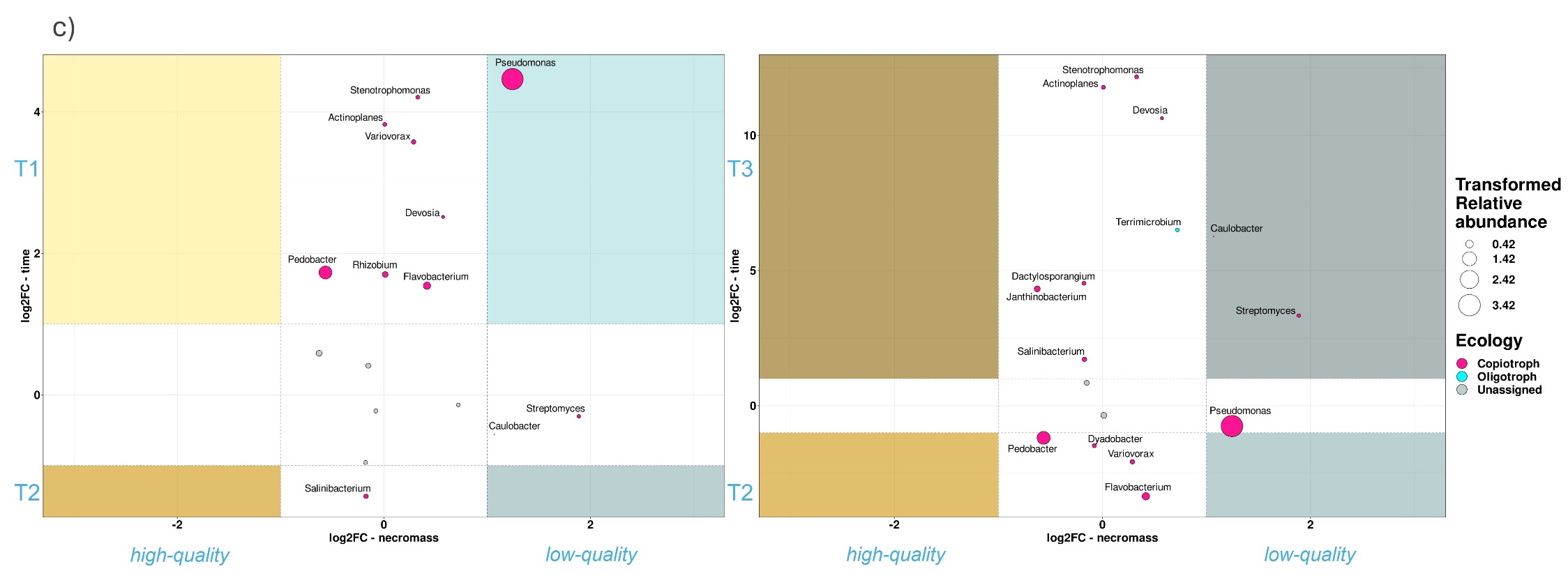


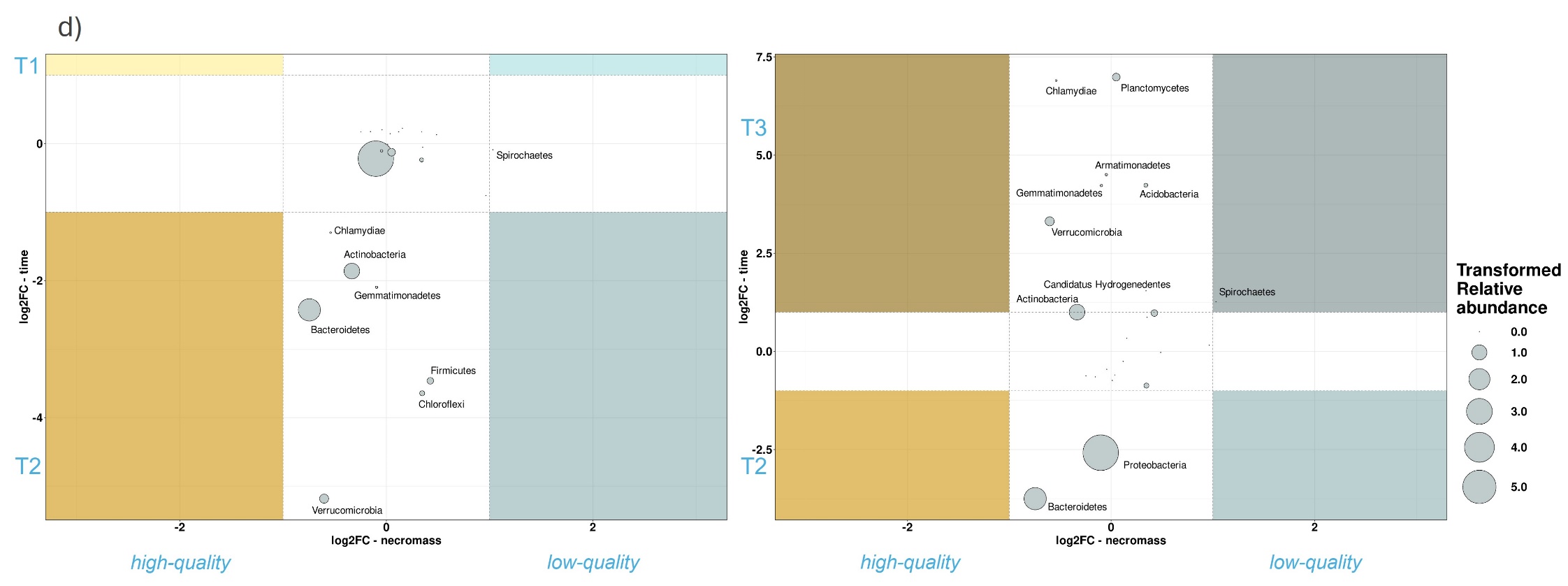

Supplement: Supplementary file 1 — Additional file1 [file 40793_2025_730_MOESM1_ESM.docx]
